# Supplementary material for: Metabolites Produced by the Oral Commensal Bacterium Corynebacterium durum Extend the Lifespan of Caenorhabditis elegans via SIR-2.1 Overexpression
Source: Int J Mol Sci. 2020 Mar 23;21(6):2212. doi: 10.3390/ijms21062212 (PMC7139712; doi:10.3390/ijms21062212)
Supplement: Supplementary file 1 [file ijms-21-02212-s001.pdf]

Figure No.	Contents	Page
Figure S1	^{1}H NMR spectrum of N-APEA isolated from *Corynebacterium durum*	S3
Figure S2	^{1}H NMR spectrum of N-ATRA isolated from *Corynebacterium durum*	S4
Figure S3	^{1}H NMR spectrum of TRA isolated from *Corynebacterium durum*	S5
Figure S4	^{1}H NMR spectrum of TA isolated from *Corynebacterium durum*	S6
Figure S5	^{1}H NMR spectrum of synthesized N-APEA	S7
Figure S6	^{1}H NMR spectrum of synthesized N-ATRA	S8
Figure S7	^{1}H NMR spectrum of synthesized N-ATA	S9
Figure S8	DPPH radical scavenging activity of the three monoamines TA, PEA, and TRA and their *N*-acetyl derivatives.	S10

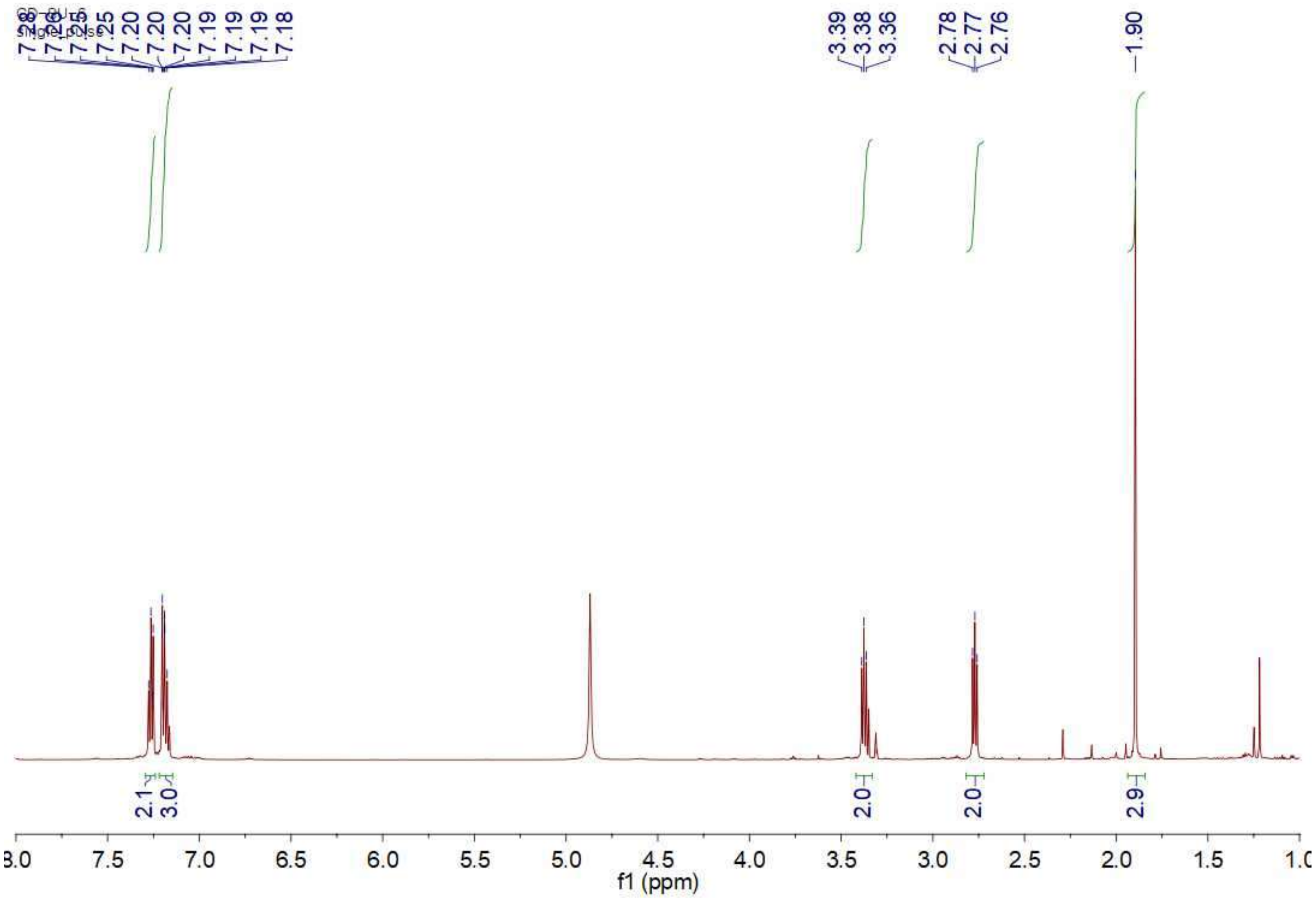

Figure S1. ^{1}H NMR spectrum of N-APEA isolated from *Corynebacterium durum.*

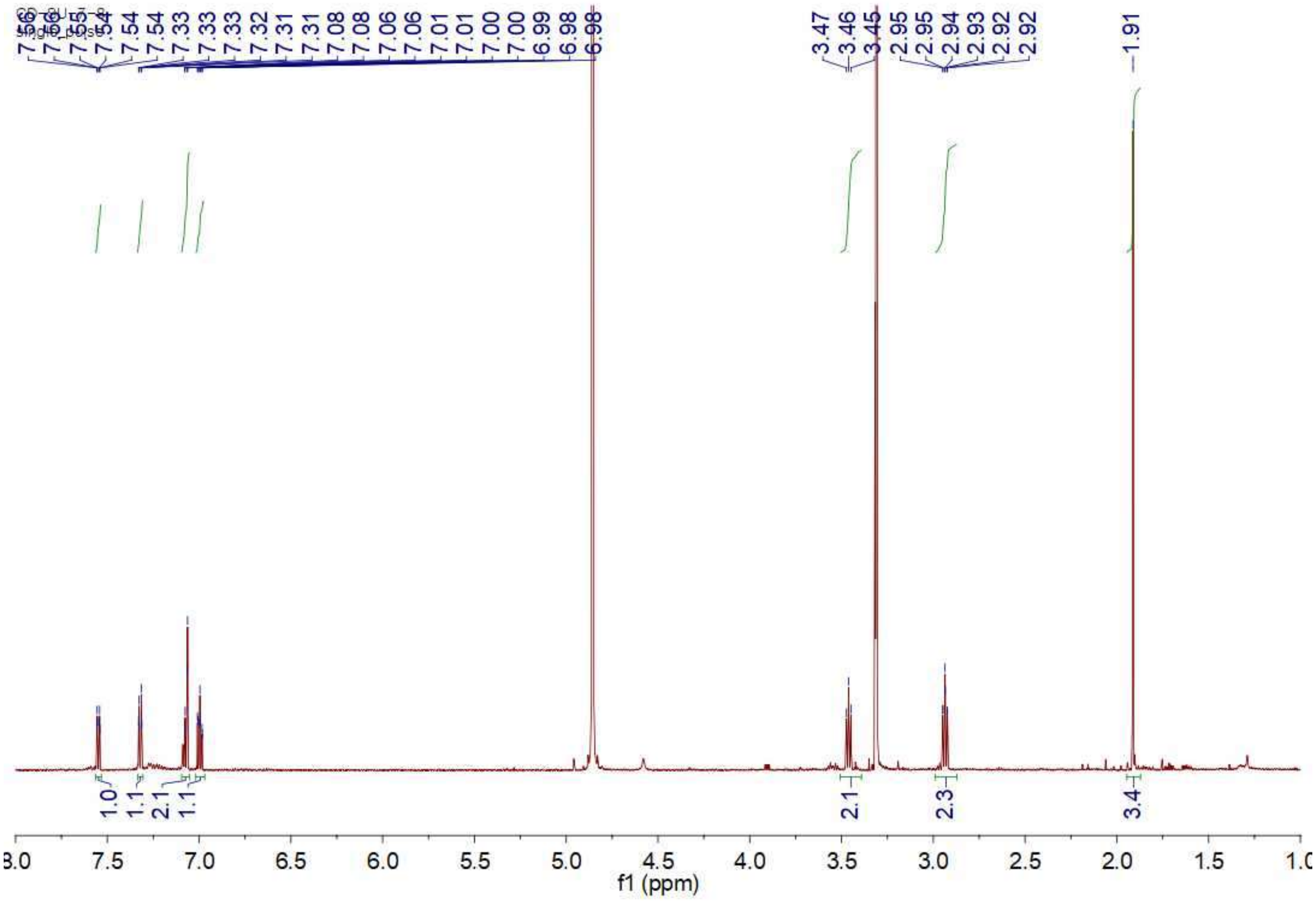

Figure S2. ^{1}H NMR spectrum of N-ATRA isolated from *Corynebacterium durum*

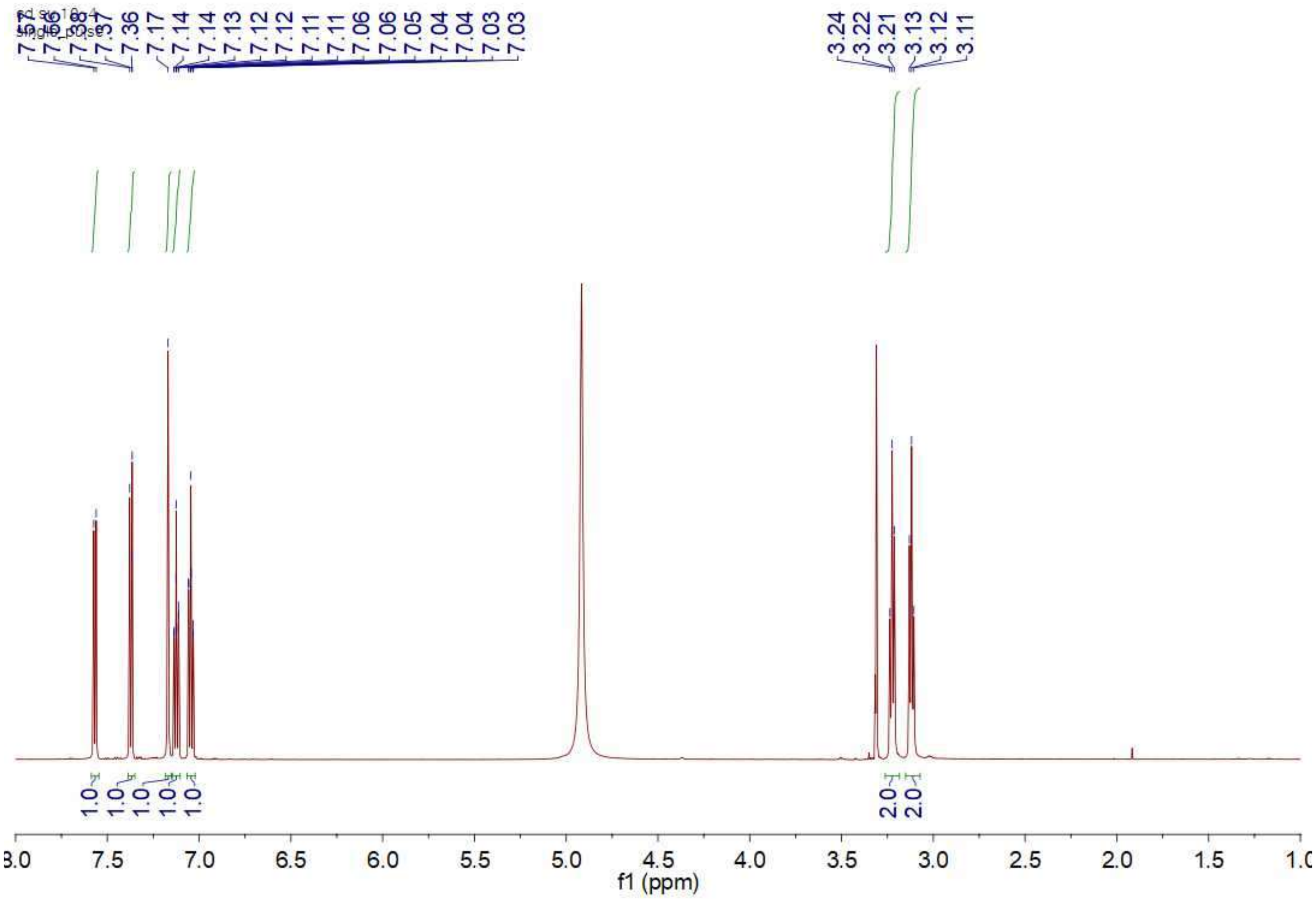

Figure S3. ^{1}H NMR spectrum of TRA isolated from *Corynebacterium durum*

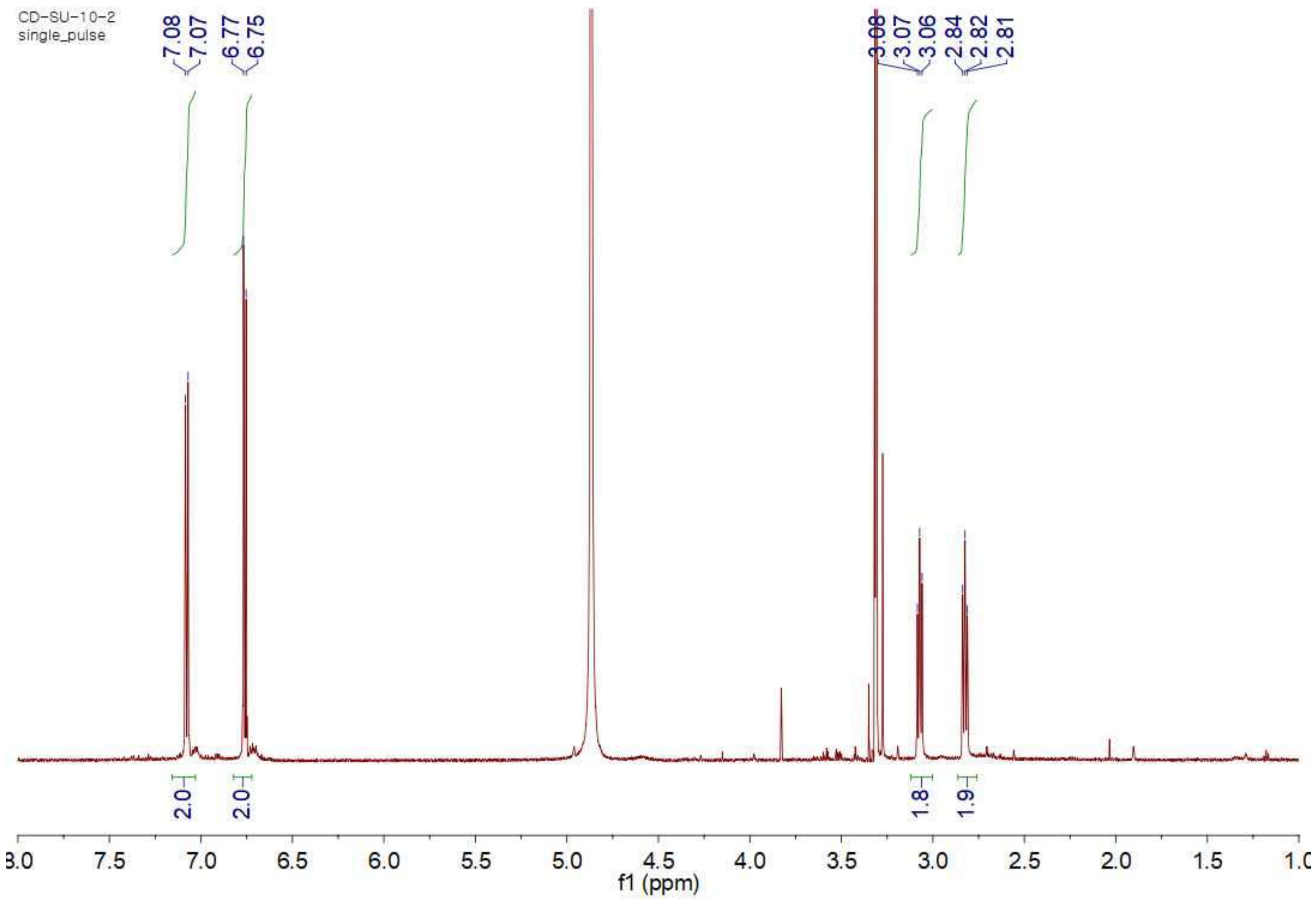

Figure S4. ^{1}H NMR spectrum of TA isolated from *Corynebacterium durum*

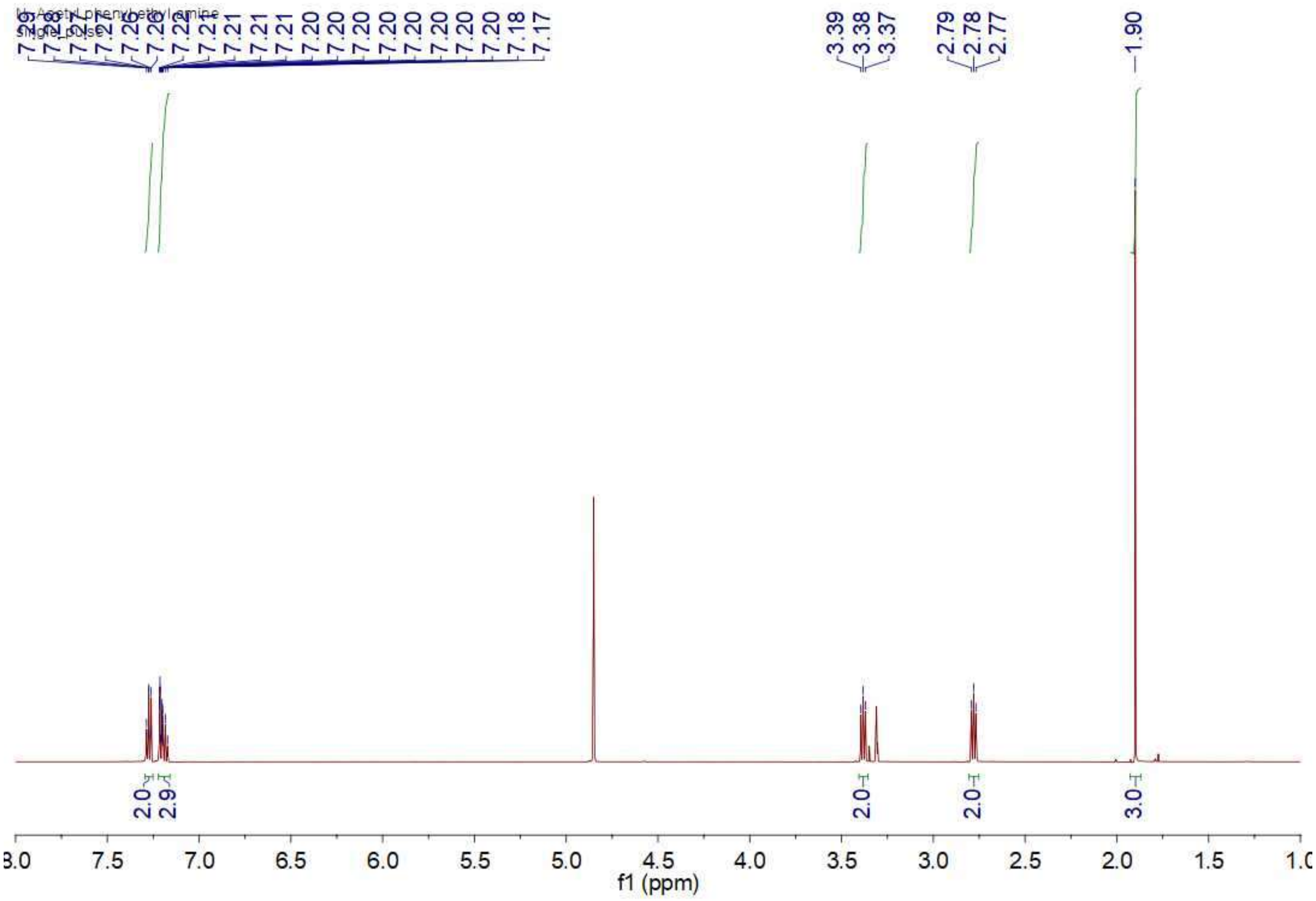

Figure S5. ^{1}H NMR spectrum of synthesized N-APEA.

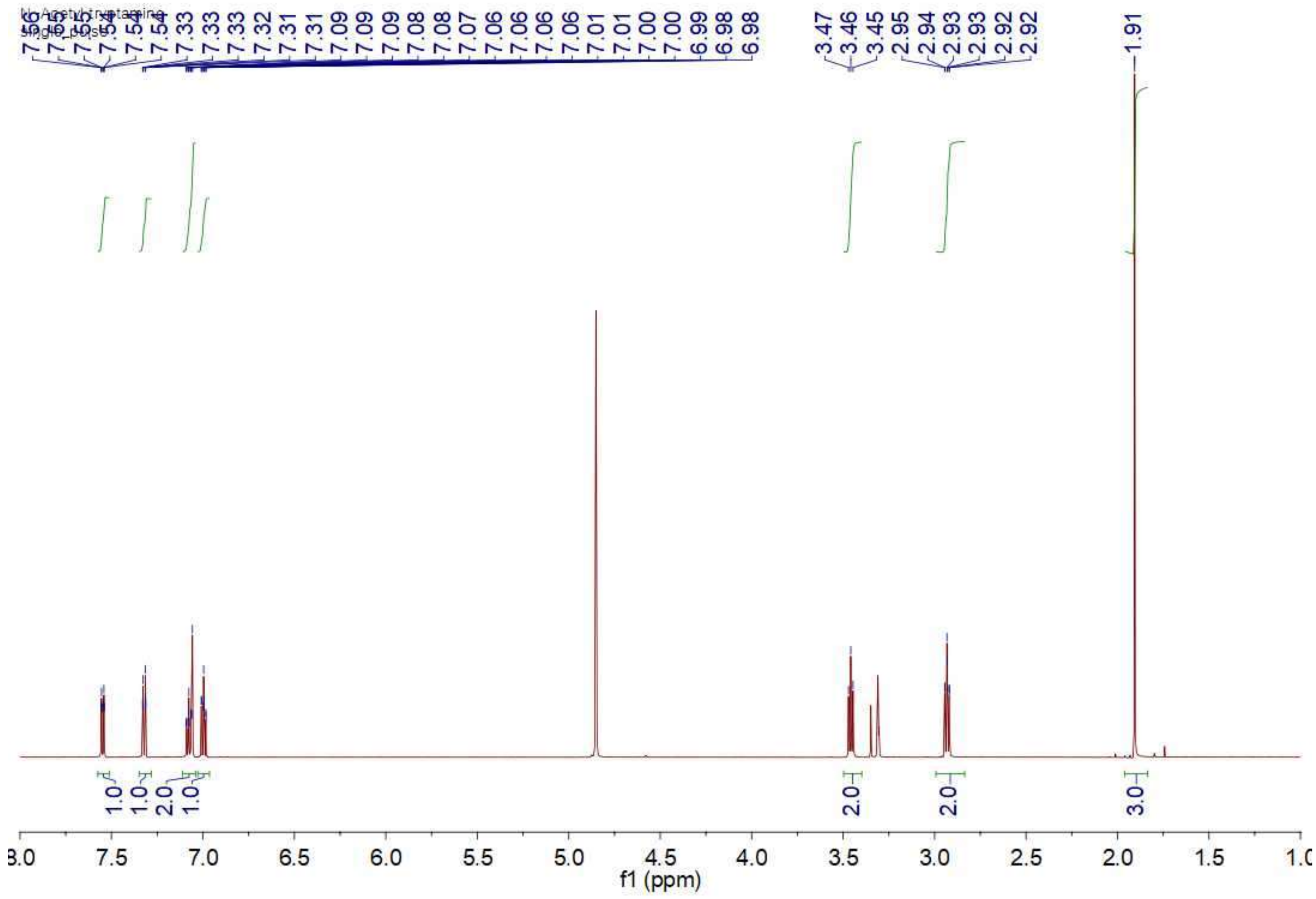

Figure S6. ¹H NMR spectrum of synthesized N-ATRA.

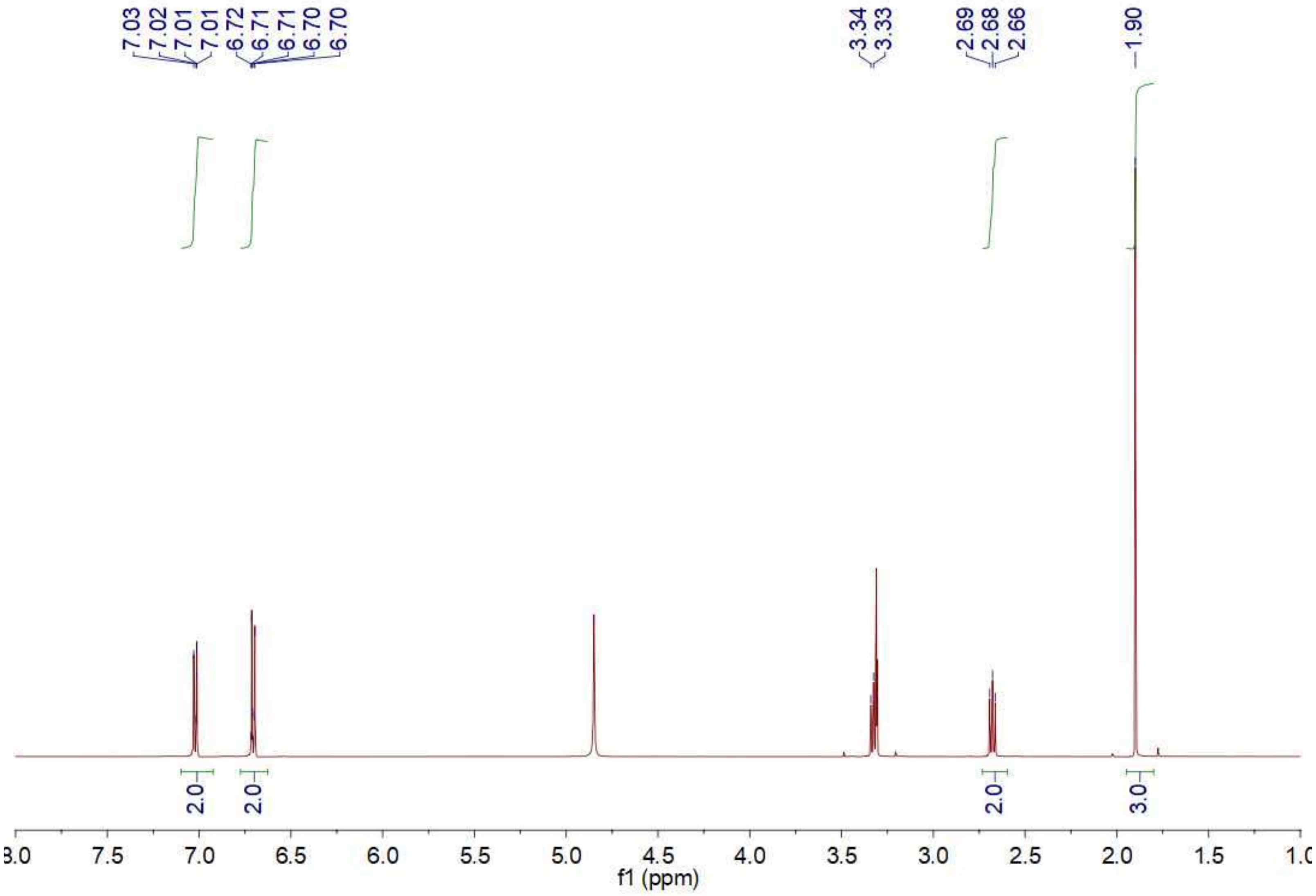

Figure S7. ^{1}H NMR spectrum of synthesized N-ATA.

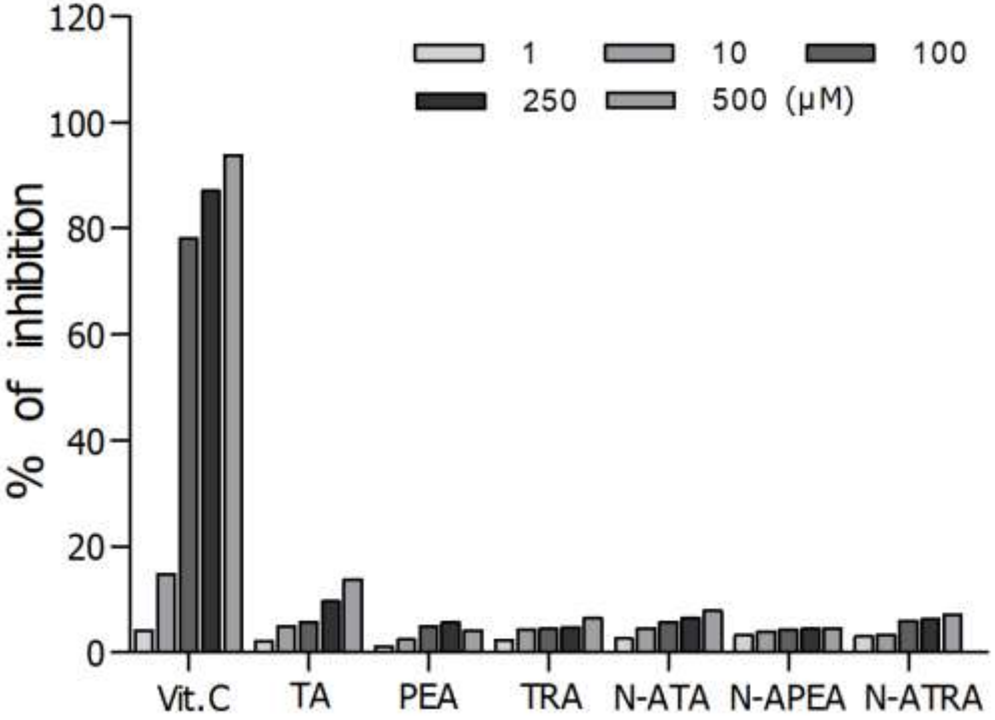

Figure S8. DPPH radical scavenging activity of the three monoamines TA, PEA, and TRA and their *N*-acetyl derivatives (Vitamin C was used as a positive control).
